# Behavioral and emotional adaptations of obese and underweight students in response to the COVID-19 pandemic

**DOI:** 10.1057/s41599-022-01334-x

**Published:** 2022-09-21

**Authors:** Mahdi Rezapour, F. Richard Ferraro, Sabrina Alsubaiei

**Affiliations:** 1 Independent Researcher, Marlborough, USA; 2 Department of Psychology, University of North Dakota, Grand Forks, ND 58201 USA

**Keywords:** Psychology, Health humanities

## Abstract

Previous studies have investigated the effects of COVID-19 on the general population of college students. However, research evaluating the complex behavioral and psychological impact of the pandemic on both obese and underweight students is currently limited. We used data from a survey conducted from March to April 2020 across 2534 students enrolled in seven US universities. We examined whether the associations between taking various behaviors and being obese and underweight students are unidimensional, or varies based on various negative emotions, and their sociodemographic characteristics. Also, we checked whether emotions of obese and underweight groups are impaired, which consequently might impact taking various cautionary behaviors. The results highlight complex relationships between being obese and underweight students and other considered variables. For instance, despite the associated risk, it was found that obese students are associated with less fear, guilt and irritability due to the pandemic. However, those associations vary based on factors such as level of educations. In addition, while obese students are less likely to avoid a large group of people, the impact changes based on gender. Lack of precautions and emotions is despite the increased risk of hospitalization and death associated with obese and underweight groups. Finally, it was found that there are negative and stable associations between higher social class, income, and the lower likelihood of being obese or underweight. Significant differences and similarities were also found across factors to obese and underweight students.

## Introduction

Past studies highlighted that the obese are at higher risk of hospitalization during the pandemic. For instance, during the COVID-19 pandemic, across individuals being impacted by COVID-19, the obese are almost twice as likely to need treatment in ICU, compared with normal-weight patients (Docherty et al., 2020). That impact has been linked to the impaired immune function of the obese (Tanaka et al., 2001).

A question might be raised why the emphasis is also given in this study to the underweight, in addition to the obese. To highlight the importance of both under- and overweight individuals, a U-shaped infection rate was observed across obese and underweight individuals (Dobner & Kaser, 2018). Similarly, another study proposed a J-shape relationship between Body Mass Index (BMI) and COVID-19 mortality, highlighting that both underweight and obese patients have a higher mortality rate than those with normal weight. In terms of prevalence, while 10% of adults are underweight globally, about one-third of them are obese (Huang et al., 2021). Despite the health concerns associated with being underweight, less research has studied that group.

Studying obese and underweight people’s emotions are especially important as the negative emotions have been described as one of nature’s crucial survival mechanisms (Dhabhar, 2014), and it is expected that mechanism to be impaired for those students. Here the emotional impairment indicates that individuals are having difficulty experiencing and expressing their emotions. For instance, obese people might take the pandemic less seriously, and they might not take necessary precautions, compared with the normal population, due to a possible reason of having impaired emotions during the pandemic.

During the pandemic, having impaired emotions might result in dire consequences due to the lack of taking precautionary behavioral actions. For instance, at the time of the pandemic, stress might motivate students to stay away from a large gathering and thus could serve as a means of protection. As we hypothesized that the non-normal-weighted conditions are associated with experiencing lack of various negative emotions, in the next few paragraphs we will discuss research on the phenomena of impaired emotion.

The association between emotion impairment and obesity was measured in a past study (Pinna et al., 2011). The results highlighted that impaired emotion is significantly more frequent across obese, compared with the normal group. On the other hand, limited studies have examined both underweight and obese individuals to highlight their possible impaired emotions, compared with the healthy weighted individuals.

For instance, a study considered both groups, highlighted that while increased age, higher educational status, and higher wealth are positively associated with being obese, those are inversely associated with being underweight (Hashan et al., 2020). Also, being female, having a higher education, greater wealth, and doing fewer physical activities are associated with obesity, while lower education and lower wealth are associated with being underweight (Pengpid & Peltzer, 2020).

In another study, it was found that wealth and lifestyle are associated with underweight individuals (Little et al., 2016). Being underweight, in another work, was found to have psychological effects, e.g., getting irritated easily and having inflexible thinking, impaired concentration, difficulty in making decisions, physical effects, and withdraw socially (Fairburn, 2008).

Studying both obese and underweight students is especially important as being obese or underweight can have serious impacts on health. For instance, obese individuals are more likely to suffer from stress, and chronic conditions such as joint pain, heart disease and cancers (Trakas et al., 1999), being underweight, on the other hand, is associated with poor nutrition and infertility (Tjepkema, 2006). In addition, underweight individuals are expected to have undiagnosed diseases, and they often have an increased risk of mortality (Kelly et al., 2010).

Limited studies also focused on younger adults and students’ obesity, considering only the additive impacts of variables. While evaluating the obesity and physical activity in college students, it was found that healthy diet and physical activities are significantly lower for obese college students (Huang et al., 2003). Relationship between obesity, depression and emotional eating was evaluated in young adults, where depressive symptoms and emotional eating were more pronounced in obese adults (Lazarevich et al., 2016). Obese college students were also found to be more emotionally reactive and more likely to overeat when distress (Lowe & Fisher, 1983).

The COVID-19 pandemic provides a unique opportunity to study behavioral and emotional reactions of obese students. In this study we considered, various demographic, behavioral, and emotional characteristics to check how they are associated with obese students. Specifically, we challenged the assumption in the literature review that the impact of emotions on obese individuals is unidimensional. For instance, we examined how the associations between being an obese student and various behaviors are stable or changing based on students’ gender, income, or various negative emotions.

The research questions this study is focused on could be summarized as follows:

(1) Do associations between various emotions and behaviors, and the likelihood of being obese or underweight students vary across students with various socioeconomic characteristics, or even their parents’ educations or income,

(2) Do emotions in obese and underweight are impaired compared with others

(3) Is there any link between emotions and cautionary actions, and is the impairment observed for cautionary actions as well, and do the associations stable or interactive, varying based on other factors,

(4) Do significant differences exist across factors to the likelihood of being obese and underweight students.

## Methods

### Study design

Data were collected from a published study across 2,534 college students at seven large public universities in the U.S., during the early stages of the COVID-19 pandemic (Browning et al., 2021). The original study was not designed for obese students, but that group was filtered based on their weight and their height. Human subject approval was awarded at each institution including Clemson, North Carolina, and Utah university in the original study, where the method was carried out in accordance with relevant guidelines and regulations. It should be noted that the data were collected in spring 2020, when the first and most severe real lockdown occurred in the U.S. Several points are worth mentioning here regarding the sources of the survey items, which the next few paragraphs will outline.

Negative emotions, such as being afraid, irritable, guilty, and sad, were based on the development of the positive and negative affect schedule (PANAS) (Watson & Clark, 1994). In the source article, participant burden was minimized by using items answered on the visual analog scale (VAS) (Jiang et al., 2016). These questions related to how much time students thought about the pandemic, where a concept derived from the eating disorder literature (Babbitt et al., 1990).

Some of survey items measured the concepts such as negative emotion states, preoccupation with COVID-19, feeling stressed, worry, and time demands. For coming up with the items, the original study examined studies of other large-scale disasters (i.e., the World Trade Center terrorist attacks on September 11, 2001), which are associated with psychological impacts on the general population (Neria et al., 2008).

Based on the past research, numerous risk factors such as race, gender, age, perceived social class, academic status, parental education, and relative family income were included in the survey as well. Socioeconomic status (SES) variables included seven questions on class, parental education, and relative family income (Rubin & Kelly, 2015). To measure academic status, the respondents were asked whether they were in pursuit of an undergraduate or graduate degree.

General health from poor to excellent measured (Centers for Disease Control & Prevention, 2018) and BMI were calculated as potential confounding factors for COVID-19 (Xiao et al., 2020). BMI was calculated from self-reported students’ height and weight. Time spent on screens or performing exercise were included as possible lifestyle-related factors (Tudor-Locke et al., 2010). Here, the lifestyle factors related to time outdoors included the number of hours in the last day spent in parks, and on greenway trails. “Screens” included using a smartphone, computer, watching television, or online gaming.

Likert-type response scales with strongly agree-strongly disagree anchors ranged from 1 and 5 or 1 and 7 were used for the majority of questions. The lowest values for ordinal and binary variables were considered as the reference categories. The response variable was the amount of stress due to the COVID-19 pandemic that the students experienced. Negative emotions based on the PANAS, on the other hand, were measured on a 1–100 response scale.

Time demands were measured by the original study (Brown et al., 2016). Those questions were developed from survey prompts in the eating disorder literature (Babbitt et al., 1990). Specifically, the original study asked to what extent respondents believed they spent time on the pandemic. For that, a 5-point Likert-type agree-disagree response scale was used. As a potential risk factor, we asked from the respondents if they know people who were diagnosed with the virus, e.g., someone in their family or someone in their community (Ghafoori et al., 2009).

Three categories were considered for the response including underweight, obese, and normal-weighted students as a reference category. The students were asked to report their height and weights and the BMI was estimated from those values. The BMI is defined as $\frac{{{\mathrm{weight}}\,\left( {kg} \right)}}{{{\mathrm{height}}^2\left( {m^2} \right)}}$, which is an index, correlating with the body fat content (Manson et al., 1987). In this study, underweight and obese was defined as people with BMI < 18.5 and BMI > 30, respectively (World Health Organization, 1997). Inclusion of various negative emotions and health-related factors are especially important as it has been found that obesity is associated with various psychological problems such as depression, lower quality of life, and lower self-esteem (De Niet & Naiman, 2011).

In addition to considering cautionary behaviors, understanding various negative emotions is especially important as they can impact obese students’ behaviors. For instance, enlarging the food intake, in which food is used as a means of coping with psychological problems, is common for the obese (Kaplan & Kaplan, 1957). Table 1 present summary statistics of important predictors across all categories, being separated across each category. For all categories, minimum and maximum are provided, while for each category lower and upper confidence interval (CI) are provided.

**Table 1:** Descriptive summary of significant predictors in the analysis.

Attributes	Type	Mean	Var	Min/LCI	Max/HCI
*Response*					
BMI, *BMI* ≤ *18.5*, 112 (5%)	Categorical	2.76	0.367	1	3
*BMI* ≥ *30*, 210 (9%)					
*Normal BMI*, 1932 (86%)					
*Students’ characteristics*					
Health in general, *poor (1)*^*a*^*, average (2), good (3) very good (4) excellent (5)*	Discrete	3.34	1.011	1	5
Obese		3.14	1.059	3.096	3.176
Underweight		2.67	0.870	2.630	2.712
Normal		3.4	0.990	3.362	3.439
Age, *18–24 years old, versus others*	Categorical	0.77	1.792	0	1
Obese		0.89	0.100	0.875	0.900
Underweight		0.64	0.231	0.622	0.660
Normal		0.78	0.174	0.759	0.792
Gender, *male, versus female*	Categorical	0.61	0.236	0	1
Obese		0.773	0.170	0.757	0.789
Underweight		0.619	0.231	0.600	0.638
Normal		0.597	0.230	0.578	0.616
*Students’, and parents’ socioeconomic characteristics*					
Student’s level of education, *undergrad (0), graduate (1)*	Categorical	0.20	0.158	0	1
Obese		0.136	0.118	0.123	0.149
Underweight		0.253	0.189	0.236	0.270
Normal		0.194	0.156	0.179	0.209
Income relative to others in the US, *well below average (1), slightly below average (2), average (3), slightly above average (4), well above average (5)*	Discrete	3.34	1.227	1	5
Obese		2.98	1.332	2.935	3.025
Underweight		3.44	0.973	3.404	3.481
Normal		3.337	1.213	3.293	3.380
Social class, *working class (1), lower middle-class (2), middle-class (3), upper middle-class (4), upper class (5)*	Discrete	2.85	1.023	0	5
Obese		2.382	1.088	2.341	2.423
Underweight		2.967	0.741	2.933	3.00
Normal		2.865	1.024	2.825	2.905
Father education, *less than high school (1), high-school graduate (2), some college (3), 2-year degree (4), 4-year degree (5), professional degree (6), doctorate (7)*	Discrete	4.56	2.662	1	7
Obese		4.065	3.140	3.996	4.134
Underweight		4.810	2.061	4.7543	4.866
Normal		4.583	2.606	4.520	4.646
Mother education, *less than high school (1), high-school graduate (2), some college (3), 2-year degree (4), 4-year degree (5), professional degree (6), doctorate (7)*	Discrete	4.53	2.220	1	7
Obese		4.167	2.722	4.102	4.231
Underweight		4.551	1.813	4.499	4.604
Normal		4.549	2.208	4.4914	4.607
*Negative emotions*					
How guilty do you feel when you think about COVID-19?, *not at all (0,), extremely afraid (100)*	Discrete	24.54	658.831	0	100
Obese		23.81	775.689	22.726	24.896
Underweight		23.90	671.047	22.90	24.92
Normal		24.44	644.16	23.46	25.44
How afraid do you feel when you think about COVID-19?, *not at all (0,), extremely afraid (100)*	Discrete	50.51	742.640	0	100
Obese		52.92	859.710	51.77	54.06
Underweight		55.27	710.490	54.23	56.31
Normal		50.00	728.870	48.95	51.06
How irritable do you feel when you think about COVID-19?, *not at all (0,), extremely guilty (100)*	Discrete	59.12	792.710	0	100
Obese		60.51	751.910	59.44	61.58
Underweight		58.37	914.600	57.19	59.55
Normal		59.37	789.160	58.28	60.47
How sad do you feel when you think about COVID-19?, *not at all (0,), extremely guilty (100)*	Discrete	61.12	725.580	0	100
Obese		4.41	3.530	4.34	4.49
Underweight		4.38	3.364	4.31	4.45
Normal		4.33	2.984	4.26	4.40
*Rational and irrational precautionary actions*					
COVID-19 precautionary actions: limit exercising at home, *never (1), rarely (2), sometimes (3), most of the time (4), always (5)*	Discrete	2.3	1.515	1	5
Obese		2.52	1.930	2.46	2.57
Underweight		2.38	1.331	2.34	2.43
Normal		2.29	1.50	2.25	2.34
COVID-19 precautionary actions: Avoiding large group of people, *never (1), rarely (2), sometimes (3), most of the time (4), always (5)*	Discrete	4.72	0.411	1	5
Obese		4.76	4.741	4.78	4.76
Underweight		4.78	4.753	4.80	4.78
Normal		4.74	4.680	4.73	4.71
Think a lot about COVID-19, *strongly disagree (1), Disagree (2), Somewhat disagree (3), Neither agree nor disagree (4), Somewhat agree (5), Agree (6), Strongly agree (7)*	Discrete	4.36	3.030	1	7
Obese		4.41	3.540	4.34	4.49
Underweight		4.381	3.356	4.311	4.45
Normal		4.330	2.981	4.263	4.398

^a^Highlights variables category.

### Statistical methods

In this study, the multinomial logit model (MNL) was used due to the categorical nature of the response. The iteratively reweighted least square (IRLS) was solved by means of the vectorized generalized linear model (VGLM) for maximization of the likelihood through the minimization of deviance. The *jth* linear predictor of $\eta _i = g_j\left( {\theta _j} \right)$, in generalized linear model (GLM), is written as:

1 $$\eta _j = \eta _j\left( x \right) = \mathop {\sum }\limits_{p = 1}^P \beta _{jp}x_p$$

where *p* is number of parameters to be estimated, and *j* is number of response category. The above formula is based on the combination of all explanatory variables in a single matrix. For iteration *a*, we have $\eta ^{(a)} = X_{{\mathrm{VLM}}}\beta ^a$.

So (Yee, 2010):

2 $$\left(\begin{array}{l}\eta_1\left(x_{i}\right)\\ \vdots \\ \eta_{j}\left(x_{i}\right)\end{array}\right) = \left({\begin{array}{ccc} {\beta _{(1)1}} & \cdots & {\beta_{(1)p}} \\ \vdots & \ddots & \vdots \\ {\beta_{(j)1}} & \ldots & {\beta_{(J - 1)p}}\end{array}}\right)x_i$$

The model parameters estimations based on the IRLS includes a creation of matrices of transformed response as $z^{(n)} = \eta _i^{(n)} + \left( {W_i} \right)^{(n)^{ - 1}}u_i^{(n)}$, where $\left( {u_i} \right)_j = \frac{{\partial \ell _i}}{{\partial \eta _j}}$ is the score vector for *jth* element, and $\left( {W_i} \right)_{jk} \,=\, \frac{{ - \partial ^2\ell _i}}{{\partial \eta _j\partial \eta _K}}$, where $(W_i)_{jk}$ measures the amount of information each observation carries. Transformed or working response could be written as:

3 $$z_{n - 1} = \beta _nX_{{\mathrm{VLM}}} + \varepsilon _{n - 1}$$

In summary, the generalized least square (GLS)’ system of equations will be converted into the ordinary least square (OLS), by pre-multiplying both side of the equation by the Cholesky decomposition, which standardize the error terms and remove the correlations across them. The use of Cholesky decomposition is to obtain the *U* matrix, which is the square root of the weight. The weight itself is written as $W = U^\intercal U$. Now matrix of *U* will be used for obtaining the OLS by multiplication of the left- and right-hand sides of Eq. 3 by *U*. So, Eq. 3 turns into:

4 $$z_{a - 1}^{ \ast \ast } = X_{VLM_{a - 1}}^{ \ast \ast }\beta _a + \varepsilon _{a - 1}^{ \ast \ast }$$

Now the above equation could be solved by the OLS.

In this study, a single variable is processed by orthogonal polynomial by the help of QR matrix decomposition, where *Q* is the orthogonal matrix and *R* is a triangular matrix. Despite obtaining a better fit in case of processing more variables, no more variable was processed due to a lack of interpretability of obtained variables by the process.

The QR decomposition is equivalent to Gram Schmidt orthogonalization, in which case, it builds a sequence of orthogonal polynomials that approximate the function with minimal least-squares error. The polynomial with degree of freedom (DF) of 3 was used for the parameter of students’ mothers’ education.

The process could be summarized as getting the variable to the power up to the degree of freedom and then using the main process of entering and normalizing the variables and getting QR decomposition to make the created columns more stable.

We considered the pairwise interaction terms across various predictors. Considering the interaction terms between emotions and behaviors is especially important as the associations between behaviors and being obese or underweight might not be stable. Considering the interaction terms helps to better understand the real underlying impacts of emotions in shaping behaviors. The model is implemented with VGAM package in R (Yee, 2010). In Table 1, we include the confidence interval, which is estimated from the Eq. 5 as

5 $$x \pm t_{\frac{\alpha }{2},N - 1}S_{\bar x}$$

where $x \pm t_{\frac{\alpha }{2}}$ is to form an area of $\frac{\alpha }{1}$, for each tail of t-distribuation, and $S_{\bar x}$ is the standard error of the mean.

## Results

This section is presented in two subsections of main and interaction terms. The *p*-value < 0.1 was considered as a cutting point for keeping variables in the model.

### Main effects

Across all considered variables, we found that four variables have only main effects, which this subsection will discuss.

Higher subjective health is associated with lower likelihood of being underweight, $\hat \beta _{{\mathrm{underweight}} - {\mathrm{Subjective}}\,{\mathrm{health}}} = - 0.29$, and obese, $\hat \beta _{{\mathrm{obese}} - {\mathrm{Subjective}}\,{\mathrm{health}}} = - 0.66$, respectively, with a higher negative impact being related to the obese. Our results show that while higher age is negatively associated with being obese, $\hat \beta _{{\mathrm{obese}} - {\mathrm{Age}}} = - 0.46$, it is positively associated with underweight students, $\hat \beta _{{\mathrm{Age}}} = 0.74$.

In addition, results highlighted that the higher social class of students, $\hat \beta _{{\mathrm{obese}} - {\mathrm{Social}}\,{\mathrm{class}}} = - 0.30$ is associated with a lower likelihood of being obese. Regarding parents’ education, we found higher father’s education to be associated with a lower likelihood of being obese, $\hat \beta _{{\mathrm{obese}} - {\mathrm{Father}}\,{\mathrm{education}}} = - 0.09$.

### Interaction terms

The interaction terms between most predictors were considered and found to be important. The majority of interaction terms include various negative emotions. It is clear, for interpreting the interaction term both the interaction and the main effects should be considered. Also, if either of the main effect’s estimates or interaction terms is significantly larger, that value makes a more significant impact on the interaction term.

### Avoiding large group due to COVID-19× Gender

The results show that the association between taking the precautionary action of avoiding large groups due to COVID-19 and being obese is not stable and varies based on the gender of students. Regarding the obese students, as the main effect of gender, $\hat \beta _{{\mathrm{obese}} - {\mathrm{Gender}}} = 3.62$, is much larger than the main effect $\hat \beta _{{\mathrm{obese}} - {\mathrm{Avoid}}\,{\mathrm{large}}\,{\mathrm{group}}} = 0.57$ and also the interaction term, $\hat \beta _{{\mathrm{obese}} - {\mathrm{Interaction}}} = - 0.74$, gender has the most important association.

### Being afraid due to COVID-19× Relative income

Similarly, we found that while there is a negative association between both obese students and being afraid of COVID-19, this association is different across obese students with various relative income. For both groups, the highest coefficient estimate is related to the income. For instance, higher income is negatively associated with being obese, where $\hat \beta _{{\mathrm{obese - Afraid}}} = - 0.01,\hat \beta _{{\mathrm{obese}} - {\mathrm{Relative}}\,{\mathrm{income}}} = - 0.12,\,{{{\mathrm{and}}}}\,\hat \beta _{{\mathrm{obese}} - {\mathrm{Interaction}}} = 0.005$.

### Feeling guilty due to COVID-19× limit exercising at home due to COVID-19

Our results highlighted that while there is a negative association between being obese and limiting exercise at home, feelings of guilt mitigate the association slope. It should be noted that only the interaction across the terms for obesity was found to be important. For that group, the point estimates of main effects are much higher than the estimate of the interaction term, where $\hat \beta _{{\mathrm{obese}} - {\mathrm{Limit}}\,{\mathrm{exercise}}\,{\mathrm{at}}\,{\mathrm{home}}} = - 0.04,\,\hat \beta _{{\mathrm{obese}} - {\mathrm{Guilty}}} = - 0.01,\,\hat \beta _{{\mathrm{obese}} - {\mathrm{Interaction}}} = 0.004$.

### Feeling irritable due to COVID-19× think a lot about COVID-19

The next interaction term is related to feeling irritable, where point estimates $\hat \beta _{{\mathrm{underweight}} - {\mathrm{Irratible}}} = - 0.02,\,\hat \beta _{{\mathrm{underweight}} - {\mathrm{Think}}\,{\mathrm{alot}}\,{\mathrm{about}}\,{\mathrm{Covid}}} = - 0.28,\hat \beta _{{\mathrm{underweight}} - {\mathrm{Interaction}}} = 0.004$ are for underweight only. The result indicates that being underweight is associated with feeling less irritable and thinking less about the pandemic. In general, this result somehow is in line with the previous study that underweight students are more likely to get irritated easily (Fairburn, 2008).

### Feeling irritable due to COVID-19× Education’s levels of students

The term is also found to be important only for obese students. From Table 2, it is clear that the main effects are much stronger than the interaction term, where point estimates are $\hat \beta _{{\mathrm{obese}} - {\mathrm{Feeling}}\,{\mathrm{irratible}}} = - 0.01,\,\hat \beta _{{\mathrm{obese}} - {\mathrm{Level}}\,{\mathrm{of}}\,{\mathrm{education}}} = - 0.70,\hat \beta _{{\mathrm{obese}} - {\mathrm{Interaction}}\,{\mathrm{term}}} = 0.002$. In general, being obese is associated with lower level of education and lower feeling of irritability.

**Table 2:** Associated factors to underweight and obese.

Attributes	Obese, BMI ≥ 30			Underweight, BMI ≤ 18.5		
	Est.	SE	*p*-value	Est.	SE	*p*-value
*Students’ characteristics*						
Health in general	−0.66	0.079	<0.005	−0.29	0.099	<0.005
Age, 18–24 years old	−0.46	0.176	0.01	0.74	0.313	0.02
Gender	3.62	0.968	<0.005	−2.23	1.412	0.1
*Students’, and parents’ socioeconomic characteristics*						
Education’s level of students	−0.72	0.441	0.1	0.30	0.547	0.6
Relative income to others in the US	−0.12	0.146	0.4	−0.39	0.200	0.05
social class	−0.30	0.089	0.001	0.05	0.131	0.7
Father education	−0.09	0.055	0.1	0.13	0.079	0.1
Negative emotions						
How guilty do you feel when you think about COVID-19?	−0.01	0.007	0.04	−0.002	0.009	0.9
How afraid do you feel when you think about COVID-19?,	−0.01	0.008	0.3	−0.02	0.012	0.1
How irritable do you feel when you think about COVID-19?,	−0.01	0.007	0.09	−0.02	0.008	0.02
Sad	−0.01	0.004	0.03	0.0001	0.005	0.9
*Rational and irrational precautionary actions*						
Limit exercising at home due to COVID-19	−0.04	0.081	0.6	−0.01	0.113	0.9
Avoiding large group due to COVID-19	0.57	0.140	<0.005	−0.33	0.163	0.04
Think a lot about COVID-19	−0.13	0.099	0.2	−0.28	0.126	0.02
*Interaction terms*						
Avoiding large group due to COVID-19× Gender	−0.74	0.203	<0.005	0.65	0.297	0.03
Feeling afraid due to COVID-19× Relative income	0.005	0.002	0.04	0.01	0.003	0.01
Feeling guilty due to COVID-19× Limit exercising at home due to COVID-19	0.004	0.002	0.08	−0.002	0.003	0.7
Feeling irritable due to COVID-19× Think a lot about COVID-19	0.002	0.001	0.2	0.004	0.002	0.03
Feeling irritable due to COVID-19× Education’s levels of students	0.02	0.007	0.01	−0.01	0.009	0.3
Polynomial variable						
Poly (Educ_Mom, degree = 3)1:1	−0.09	4.125	0.9	−6.28	6.344	0.3
Poly (Educ_Mom, degree = 3)2:1	2.75	3.560	0.4	−12.16	5.940	0.04
Poly (Educ_Mom, degree = 3)3:1	−2.08	3.330	0.5	−15.76	5.887	0.01
Loglik = −1021, *AIC* = 2131, versus standard model loglik = −1027, AIC:2135						

**Fig. 1:**
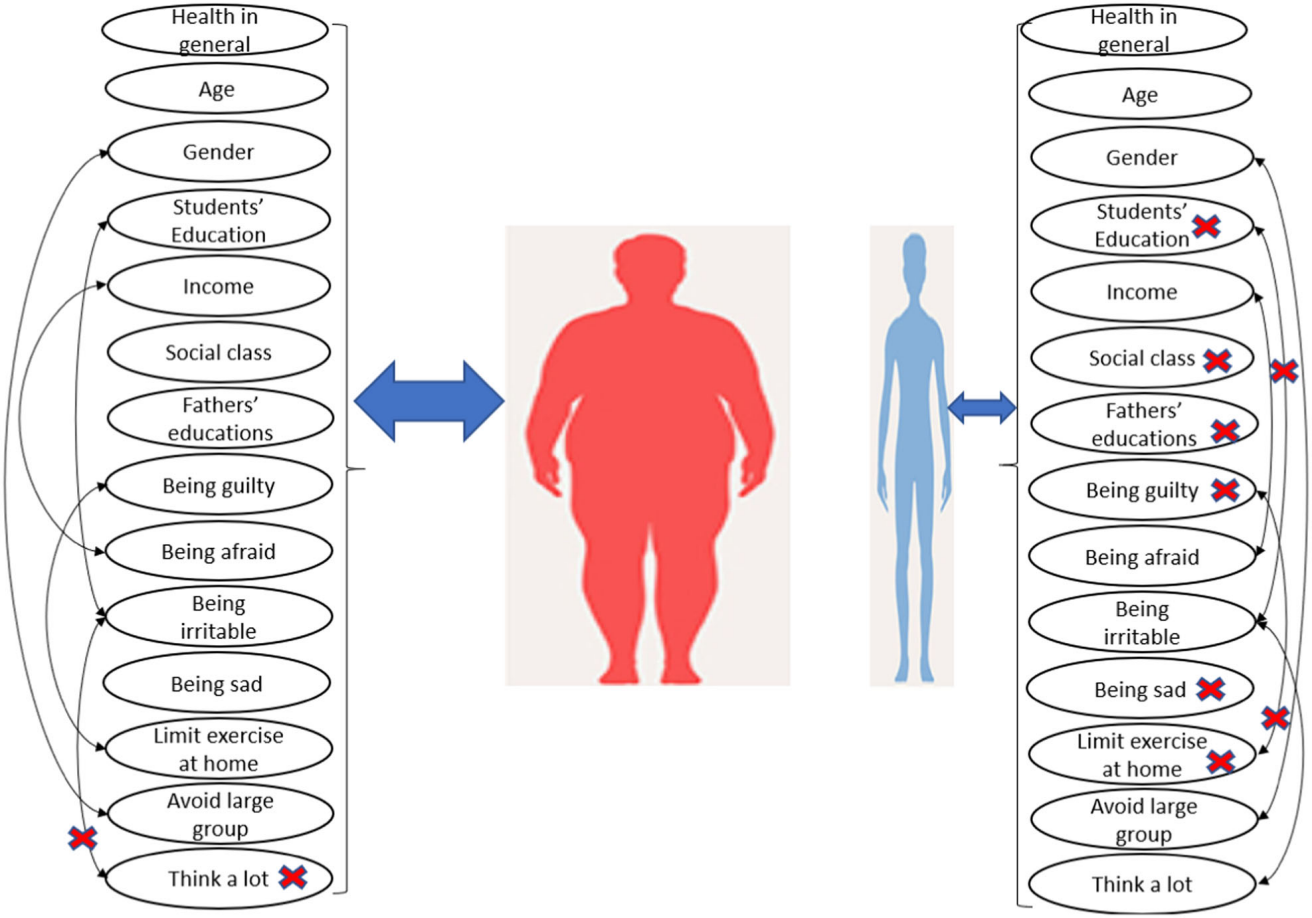
Associated factors with over-weight and underweight students, the insignificant parameters and interaction terms are crossed.

To have a better vision about associated factors of obese and underweight students, Fig. 1 is provided. Also, insignificant interactions terms are crossed in the figure for clarity.

Finally, it should be noted that using orthogonal polynomial for a single variable resulted in a slight improvement in the model fit of the model with loglik = −1021, Akaike information criterion (AIC) = 2131 versus standard model with loglik = −1027 and AIC = 2135.

To have a better vision about associated factors of obese and underweight students Fig. 1 is provided. Also, insignificant interactions terms are crossed in the figure for clarity.

## Discussion

Although associations between negative emotions, behaviors, and sociodemographic characteristics of people living with obesity have been well-established, most of past studies ignore the multiplicative associations of those predictors and obese and underweight groups of students. Studying those complex associations are especially important during a unique pandemic like COVID-19 as it expands our understanding regarding the emotional and behavioral reactions of those vulnerable groups of students.

The results highlighted that while higher father and students’ education, income and social class buffer against the likelihood of being obese students, lack of various negative emotions and taking less cautionary actions against COVID-19 contribute to the likelihood of being obese. while similarities were found between underweight and obese students, significant differences were also found across those groups, highlighting the importance of studying underweight in addition to obese students.

It was found that while feeling fear is interacting with relative income, higher feeling of fear due to COVID-19 is associated with higher likelihood of belonging to both groups. Also, higher income is associated with lower likelihood of being obese or underweight. Results are in line with the past studies showing a reverse association between obesity and higher income (Kim & von dem Knesebeck, 2018), and also between being underweight and higher income (Al Kibria et al., 2019). The association between income and negative feelings due to COVID-19 might be expected as lower income means lower control over life and consequently higher insecurity, social isolation, and various mental disorders (Marmot & Wilkinson, 2001). However, it is noteworthy the previous studies only considered the additive terms.

The subjective health was used to check its association with being obese students. The results highlighted that while there are negative association between subjective health and the likelihood of being obese and underweight, the impact is more pronounced for obese students. This result, to some extent, is in line with the previous study that there is a negative association between being obese and subjective health (Guallar-Castillon et al., 2002).

Moving to the associations between age and obese students. Our results highlighted that age has varied impacts on the two group, highlighting while higher age is positively associated with being underweight, it is negatively associated with being obese. Literature findings are not consistent. For instance, results are in line with the literature, which showed that the highest prevalence of underweight is in younger age, 15–24 years old, across women, while the middle age, 25–34 years old, has the lowest prevalence (Hashan et al., 2020). Our findings, however, differ from those in the previous study, which found that the prevalence of underweight individuals is higher across the young, while the old are more likely to be obese (Al Kibria et al., 2019).

Higher father education, social class, and income were found to be associated with a lower likelihood of being obese or underweight. That somehow could be contrasted with the literature, showing that home environment has the most important setting regarding shaping children’s physical and eating behaviors (Golan, 2006).

Specifically, the negative association between higher social class and obesity is in line with the past study, which found that there is a negative association between higher social class and obesity (Moore et al., 1962). The impact might be linked to the parents or even students’ level of educations. However, the interactions of those variables was checked but found not to be important. It is expected that there might be other confounding factors that shape the correlation between the social class with being obese students, which were not recorded by this study.

Although it is not practical to impact the social class of students, it is recommended from younger age to employ behavioral and educational programs targeting parents only, or parents and obese children to maintain a healthy lifestyle. The methodology has proven to be effective for families with obese children (Golan et al., 2006). The inclusion of parents in the program is especially important as they might act as a function of role models, shaping children’s’ behaviors in terms of physical or sedentary activity (Gustafson & Rhodes, 2006).

Higher father education was found to reduce the students’ risk of obesity. The result somehow is in line with the literature, highlighted that characteristics such as parents’ education, jobs, and even number of family members are important risk factors for weight gain and obesity (Troiano et al., 1995). The impact might be due to varied eating style, which predispose students to have certain food preference. The impact might be likelier to be shaped from the younger age, where the impact of parents might be more prevalent.

In this study, the level of education was only considered from undergraduate to graduate studies, highlighting association of minor changes of education on being obese students. The impact of this small change is expected to be due much confounding factors that were not recorded. It has been discussed that while income provides a material impact on health, education retain health, and while income takes place in adulthood, education take place in adolescence and adulthood (Geyer et al., 2006). In addition, the importance of higher education has been discussed to be associated with health literacy and behaviors (Cutler & Lleras-Muney, 2006). Policies should be designed not necessarily through persuading higher education but educating students at younger age regarding the consequences of being over-and underweight.

Despite the risk, while we found that obese students do not avoid interacting with large groups of people due to COVID-19, underweight students are more likely to avoid mixing with a large group of people, compared with normal-weight students. Similarly, past studies show that individuals with higher BMI are less concerned with the virus and taking behavioral precautions less seriously than individuals in other categories (Sutin et al., 2020). However, part of our results is against the previous study, which found a significant negative association between taking precautionary action and being underweight (Sutin et al., 2020). It should be emphasized that past studies just consider the additive effect.

Taking precautionary action of avoiding large group of people is interacting with gender. The interactive factor of gender highlighted that females are more likely to be obese, which is in line with the past research that due to behavioral and biological characteristics of women, they are more prone to be both underweight and obese compared with men (Kanter & Caballero, 2012).

It is worth reiterating the importance of studying the underweight, in addition to obese students. That is especially important as underweight individuals were found to be at higher risk compared with obese students (Dobner & Kaser, 2018). As a result, the policy makers should aim at targeting both groups simultaneously. There are both similarities and differences of associated factors across obese and underweight students, which the following paragraphs highlight few of them.

While the majority of factors and the interactions terms were significant for obese students, they were found to be not important for underweight students. For instance, students’ educations, social class and father’s education were found not to be important for underweight students. Also, differences were found in terms of taking cautionary actions against COVID-19. For instance, we found that while obese students do not avoid interacting with large groups of people due to COVID-19, underweight students are more likely to avoid mixing with a large group of people compared with normally weighted students.

In addition, although feeling of guilt was not significant for underweight students, it was for obese students. The weight-related guilt and shame were highlighted as prospective predictors of coping with obesity (Conradt et al., 2008). Although that case study and its implication differ from ours, the contrast might be still made. Our result is expected to be due to a possible impaired emotional feeling of the obese, who feel less guilt due to COVID-19 compared with normally weighted students.

Turning to limiting exercise at home. Despite the fact that limiting exercise at home does not pose a risk of being infected, normally weighted students are more likely to limit that behavior. It should be also noted the association of guilt and being obese students is interacting with limiting exercise at home due to COVID-19.

It should be noted that there are inconsistent results for the association between emotion impairment and obesity. For instance, people with higher BMI are unrelated to the concerns of the virus (Sutin et al., 2020). Also, the other study found that being obese does not unequivocally imply cautiousness (Bíró et al., 2021). However, other studies found that the obese are associated with emotional impairments (Elfhag & LUNDH, 2007). Although we cannot for certainty prove our hypothesis that the obese are associated with lack of emotions, the interaction between some emotions and different precautionary actions supports our hypothesis.

The study findings should be interpreted with caution due to the possible limitations. That is especially important as this study only focused on students in seven universities across the US. So, the scope might be limited to students in the same geographic characteristics. There is also a recall bias related to the self-reported web-based survey. In other words, this study relied upon the recall and honesty of students in question, especially questions related to the weight and height. However, it has been discussed that women and men overestimate their height and underestimate their weight (Danubio et al., 2008). The consideration is especially important as those factors were used for estimation of BMI. However, no viable method could be used to address the uncertainty regarding those factors. For future studies, random checks of some observations are recommended to assure the accuracy of the results.

Another limitation is the cross-sectional limitation of the study. That was mainly due to the unique circumstances and possible impracticality of observing the participants in varied time spans due to change in circumstances. Another noteworthy limitation was lack of validated questionnaire Again, because of the unique circumstances, it was very challenging to elongate the process of data collection due to validating the questionnaires. Another limitation is related to the populations of underweight and obese, which might not be representation of the US students. It should be noted that the data was not originally collected to evaluate those groups but the psychological impacts of COVID on students in general sense. So, the generality of the findings should be taken into consideration.

In summary, this study found that in response to the pandemic, compared with normally weighted individuals, obese individuals are associated with less adoptive behaviors, e.g., taking precautionary behaviors, and less emotional feelings, e.g., having negative emotions with lower scales. Also, the results highlighted the important role of parents on the well-being and physical health of students.

The findings point to some potential take-away that could be considered by policy makers. The results implicate while designing the educational policies for targeting obese students, the population should be stratified based on students’ characteristics such as levels of income, education, and their parents’ characteristics. That is due to identified significant interaction terms. The program should also be supported by the governmental entity to take a more holistic approach to prevent the obese and underweight in future generations.

To finalize, we showed that both obese and underweight student experience impaired levels of emotions. To our knowledge, this is one of the earliest studies that investigate both underweight and obese students while considering the complex interaction terms of negative emotions during a unique pandemic.

## Conclusions

Based on our findings, it is important to discuss the validity of our hypotheses. Our first hypothesis was about the impact of various students and parents’ demographic characteristics on the likelihood of being obese or underweight. The impacts of gender, age or even parental educations prove that the hypothesis holds true for both categories. The second and third hypotheses were about the emotional and behavioral impairments of the groups. Our finding found that especially emotional impairment across feeling less irritable, guilty, and fear for obese students, and fear for underweight students. In addition, precautionary behaviors interact with emotions, and other characteristics. For instance, while obese students are less avoiding large group, underweight care more about avoiding the large group compared with the normal-weighted students. The last point also partially answered our fourth hypothesis that there are differences across the two groups. For instance, while higher age is negatively associated with being obese, it is positively associated with underweight students.

## Data Availability

This study builds on data published earlier by the reference of Browning, et al. (2021) e0245327.
